# Intraprostatic Botulinum Toxin injection in patients with benign prostatic enlargement

**Published:** 2009-11-25

**Authors:** CP Ilie, MB Chancellor, YC Chuang, D Mischianu

**Affiliations:** * Urology Department, ‘Carol Davila’ University of Medicine and Pharmacy,BucharestRomania; ** Department of Urology, University of Pittsburgh, Pittsburgh USA; *** Division of Urology, Chang Gung Memorial Hospital Kaohsiung Medical Center, Chang Gung UniversityTaiwan

**Keywords:** botulinum toxin, prostate, BPH

## Abstract

Histological evidence of benign prostatic hyperplasia (BPH) exceeded 50% in men over 50 years of age and rose to 75% as men entered the eighth decade. Therapeutic options for BPH generally fall into one of the three categories: watchful waiting, medical treatment and surgery. Excluding watchful waiting, the other forms of intervention directed at modifying the physiologic effects of BPH with or without directly altering the prostatic mass or its configuration come with varying effectiveness and risk. Botulinum toxin (BTX–A) produce inhibition of acethylcholine release at the neuromuscular junction causes paralyzing effects and atrophy of striated as well as the smooth muscle fiber. BTX–A also causes inhibitory effects on the ganglionic and post– ganglionic fibres of autonomic nervous system inducing diffuse atrophy and apoptosis of nasal and prostate glands.

Clinical series demonstrates efficacy of BTX–A in alleviating symptoms induced by BPH. Larger randomized clinical trials studies are necessary in order to identify the mechanisms by which BTX–A affects the prostate, the ideal dose and the duration of effect. BTX–A injected into prostate appears safe and effective

## Introduction

Urinary obstruction as a result of benign prostatic disease has probably been recognized to some degree since the earliest days of medicine but Riolan probably first formalized this association, in the seventeenth century [1]. In the mideighteenth century, Morgagni [2] provided one of the earliest descriptions of benign prostatic hyperplasia (BPH) and enumerated many of the potential medical problems attendant to its development. More exact recognition of the pathologic process has been credited to Virchow in the last quarter of the nineteenth century. BPH is a highly prevalent nonmalignant enlargement of the prostate related to ageing [3– 5]. Almost 80 years ago, Randall [6] found that histological evidence of definite or probable BPH exceeded 50% in men over 50 years of age and rose to 75% as men entered the eighth decade. On the other hand, clinically important mass–producing BPH occurs in only about half (40–50%) of men with presumed histological BPH and is clinically manifested in about half of these [7]. Berry and associates [8] analysis implies that BPH is probably initiated before age 30.

Therapeutic options for lower urinary tract symptoms (LUTS) associated with BPH have expanded rapidly in the last two decades. These generally fall into one of the three categories: watchful waiting, medical treatment and surgery (including minimally invasive) [5]. Excluding watchful waiting, the other forms of intervention directed at modifying the physiologic effects of BPH with or without directly altering the prostatic mass or its configuration come with varying effectiveness and risk. Thus, there has been much interest in the development of alternative treatments such as the injection of botulinum toxin type A (BTX–A) into the prostate

## Mechanism of action and preclinical data

Botulinum toxin (BTX), the most poisonous from all poisons, was first identified in 1897 as the product of *Clostridium botulinum* [9]. From the seven immunologically distinct serotypes of BTX (A,B,C,D,E,F, and G) [10], only BTX–A and BTX type B are in clinical use [11].

Synthesized as a single–chain, inactive polypeptide (protoxin) must be cleaved, in order to be activated into a heavy chain, responsible for the specificity of each serotype and a light chain, responsible for the pharmacological action, connected to one another by a disulphide bond and non–covalent interactions. [12– 15]. Heavy chain provides binding selectively and irreversibly to ecto–receptors on the presynaptic motor nerve ending followed by internalization, and translocation of the light chain into the cytoplasm, the later preventing exocitosis of acethylcholine.

The inhibition of acethylcholine release at the neuromuscular junction causes paralyzing effects and atrophy of striated as well as the smooth muscle fiber [14]. BTX–A also causes inhibitory effects on the ganglionic and post–ganglionic fibres of autonomic nervous system inducing diffuse atrophy and apoptosis of nasal and prostate glands [16– 18].

The analgesic proprieties of BTX–A results by inhibiting neuropepdide release from nociceptive afferent C fibres, and a considerable reduction in pain [19–25]. It has been shown to inhibit the release of CGRP, substance P, glutamate, NGF, and ATP, which are mediators of painful sensation. BTX–A injection into rat proximal urethral sphincter may have significant inhibitory effects on norepinephrine release, conforming to Smith et al. [26].

Chuang et al. injected 100U Botox into the canine prostate. They observed marked atrophy and diffuse apoptosis of glands associated with decreased cell proliferation. The effect persisted for at least 3 months without any notable side effects [27]. Atrophic changes in prostate gland and vacuoles formation in smooth muscle cells of stromal tissue, and significantly reduced prostate urethral pressure response toⅣnorepinephrine and electrostimulation was also observed by Lin et al. [28].

Generalized atrophy of the glands, reduction of the total prostate volume (PV) and weight and diffuse glandular apoptosis with the TUNEL staining was also described by Doggweiler et al. on rat prostates injected with varying doses of BTX–A [29].

## Clinical data

BPH in humans is a nodular, regional growth with a variegated gross appearance resulting from the inhomogeneous and irregular mixture of glandular and stromal tissue. BPH nodules are almost always located centrally in the periurethral portion of the enlarged gland. In many instances, the nodular hyperplasia is separated by a distinct, smooth, cleavage plane from the compressed peripheral prostate that resembles a capsule. The weight of the hyperplastic tissue is highly variable, ranging from a few grams to more than 200g; no clear relationship between the size of the adenoma and the degree of bladder neck obstruction has been established.

Two main factors contribute to symptoms of BPH: excessive growth of prostatic tissue (static component) under the parasympathetic control and regulated by androgen, and increase in smooth muscle tone (dynamic component) sympathetically influenced [30]. Contraction of the autonomically controlled prostate or bladder neck smooth muscle is postulated to be a significant modifiable functional component of BPH–mediated bladder neck obstruction. BTX–A inhibits acetylcholine release at the neuromuscular and neuroglandular junction. BTX–A also blocks acetylcholine in the autonomic neurons innervating the glandular system resulting in a decreased secretion of norepinephrine release [4].

Maria et al. [31] published in 2003 the first prospective, randomized double– blind, placebo–controlled trial in 30 men with BPH (Table 1). The mean follow–up period was 19.6 months. Dose consisted in 4ml of solution (200U BTX–A). At one month respectively at two months evaluation, 11 respectively 13 patients out of 15 had symptomatic relief, the AUA (American Urological Association) score was reduced by 54% (p=0.00001) and 65% (p=0.00001) and the serum PSA level by 42% (p=0.00006), respectively 51%(p=0.00001). PV decreased by 54% (p=0.00001) and 68% (p=0.00001) at one month respectively at two–months follow–up, and the PVR (postvoid residual volume) by 60% (p=0.00001), respectively by 83% (p=0.00001) [31]. 

**Table 1:** **Abbreviations**: PI, patients improved, FU, follow–up (months); IPSS, International Prostate Symptoms Score; QoL, quality of life; Qmax, maximum urinary flow rate; PV, prostate volume; PVR, post–void residual volume; NA, not available.

Study	PSA(ng/ml)	PVR(ml)	PV (ml)	Qmax (ml/s)	QoL	IPSS	PI	FU
Maria et al; [31]	3.7 to 1.8; 51%, p=0.00001	126.3 to 21; 83%, p=0.00001	52.6 to 16.8; 68%, p<0.00001	8.1 to 15.4; p<0.00001		23.2 to 8,65%, p=0.00001	13(15)	2
Chuang et al; [18]	NA	177.6 to 24.5,86.2%, p=0.064	61.6 to 50; 18.8%, p<0.05	7..5 to 12.9; 72%, p<0.05	3.9 to 2.1; 61.5%, p<0.05	From 19 to 5,(73%, p<0.05)	8(8)	1
Chuang et al; [32]	0.8 to 0.72,NA	67.7 to 25.1; 63%, NA	19.6 to 17; 13.3%, p<0.0014	7.3 to 11.8; 39.8%, p<0.001	3.8 to 2.1; 44.7%, p<0.0001	18.8 to 8.9; 52.6%, p=0.0001	16(16)	1
Chuang et al; [33]	NA	64.2 to 35.7; 44%, p=0.3	21.1 to 18; 15%, p<0.001	7.9 to 12; 62%, p<0.001	3.9 to 2.1; 46%, p<0.001	18.7 to 9.8; 48%, p<0.001	100U	1
Chuang et al; [33]	NA	161.7 to 45.2; 72%, p=0.02	54.3 to 46.3; 15%, p<0.001	7 to 10.3; 47%,p<0.001	4.1 to 2; 51%, p<0.001	19.3 to 9.5; 51%, p<0.001	31(41)	1
Kuo; [35]	NA	243.5 to 36.8; p=0.005	65.5 to 49.6;p=0.009	7.6 to 11.6; p=0.05	4.5 to 2.1; p<0.0001	NA	10(10)	6
Park et al; [36]	2.6 to 2.4; NA	122.7 to 84.7; 34%, p<0.05	47.2 to 42; 13.1%, p<0.05	9.6 to 11.1; 15.5%, p<0.05	NA	24.3 to 16.9; 30.3%, p<0.05	39(52)	3
Silva et al; [34]	6 to 5; p=0.04	0 to 92	70 to 47; p<0.001	0 to 10.3	NA	NA	10(10)	3
Silva et al; [40]	25% decrease	55 to 82	82 to 49 (6m) then 73 (18m)	11.3 to 11.3	NA	10 to 12	11(11)	18
Brisinda et al ;[41]	6.2 to 4.8 p=0.03	92.1 to 80.3 P=0.01	54.1 to 47.2 p=0.01	8.6 to 13.1 p=0.01	NA	AUA 24.1 to 12.6 p=0.00001	41(77)	1
Brisinda et al ;[41]	6.2 to 3.0 p=0.00001	92.1 to 40.6 p=0.002	54.1 to 30.9 p=0000.1	8.6 to 16.5	NA	24.1 to 8.7 P=00001	55(77)	2
Brisinda et al ;[41]	3.0 to 3.1 p=0.7	40.6 to 27.1 p=0.03	30.9 to 26.9	16.5 to 14.5 p=0.03	NA	8.7 to 11.1 p=0.02	55+22(77)	30

Professor Chuang and Professor Chancellor, co–authors of the present review, pioneers in investigating the effects of BTX– A in prostatic tissue, have published three studies on the subject. At first they studied the effect of BTX–A in BPH on 8 men with symptomatic BPH. PV was significantly reduced from 61.6±8.7 to 50.0±5.9 ml (18.8%, p<0.05), IPSS score from 19.0±1.8 to 5.1±2.0 (73.1%, p<0.05), QoL (Quality of Life) index from 3.9±0.3 to 1.5± 0.2 (61.5%, p<0.05) and PVR from 177.6±71.7 to 24.5±4.5 (86.2%, p=0.06). Qmax increased from 7.5±1.8 to 12.9±0.5 ml (72%, p<0.05) at one month follow–up [18]. They also investigated the therapeutic effect in small prostates and reported that BTX–A improves BPH symptoms, in 16 patients with PV<30ml [32]. All patients reported subjective improvement starting at one week, achieved the maximal effect after one month and maintained it for a mean follow–up of ten months. The mean PV, IPSS score, quality of life index were significantly reduced by 13.3% (from 19.6±1.2 ml to 17.0±1.1ml, p=0.0014), 52.6% (from 18.8±1.6 to 8.9± 1.9, p<0.0001), and 44.7% (from 3.8±0.3 to 2.1±0.3, p<0.0001), respectively. The maximal flow rate was significantly increased by 39.8% (7.3±0.7 ml/sec to 11.8±0.8 ml/sec, p<0.0001) [32]. Chuang et al. also treated 41 men with 100U (N=20, for prostates<30 ml) or 200U (N=20, for prostates> 30 ml) of BTX–A [41]. LUTS and QoL indices both improved by over 30% in 31 out of 41 patients (76%). Four out of five men (80%) with urinary retention for more than one month could void spontaneously after BTX–A injection. Twelve of 41 patients (29%) did not have change of PV but 7 of these men had more than 30% improvement in Qmax, LUTS, QoL scores, suggesting that the mechanisms of LUTS relief through intraprostatic BTX–A injection may depend on the inhibitory effect on the smooth muscle tone and sensory nerve function [33]. The efficacy was maintained at 12 months. 

Twenty one men (mean age 80±2 years) with BPH, on chronic indwelling catheter for at least 3 months, with poor general condition received 200U BTX–A in a study conducted by Sylva et al. [34]. PV decreased from 70±10ml to 57±10ml(p<0.0006) at one month and to 47±7ml (p=0.03 against 1month) at three months.16 (76%) patients could resume voiding at one month, with a mean Qmax of 9.0±1.2 ml/s and PVR of 80±19ml. At three months, 17 patients (81%) voided with a mean Qmax of 10.3±1.4ml/s and PVR of 92± 24ml. PSA deceased  at three month from 6.0±1.1ng/ml to 5.0±0.9ng/ml (p=0.04) [34].

Kuo reported injection of BTX–A into transitional zone via cystoscopy [35] for benign prostatic obstruction, associated with chronic urinary retention or large residual urine. All patients had improvement in spontaneous voiding with a significant decrease at six months in PVR from 243.0±133.9 to 36.8±34.1ml (84.8%, p=0.005), PV from 65.6±19 to 49.5± 17.6ml (29.9%, p=0.009), QoL index from 4.5± 2.7to 2.1±1.9 ±53.3%, p<0.001) [35]. He also reported a decrease in Qmax from 7.6±3.9 to 11.6 (3.5ml/s ±p=0.05). The BTX–A effects appeared at one week and maintained after a mean follow–up of 9 months.

Park et al. reported symptomatic improvement in 39 out of the 52 patients transperineally injected with 100U to 300U Botox [40]. Qmax increased by 15.5% (p<0.05). The storage symptoms were improved more than the voiding symptoms. IPSS, QoL, PV, and PVR decreased by 30.3% (p<0.05), 34.3%, 13.1% (p<0.05) and 34.3% (p<0.05), respectively [36]. The follow–up period was three months.

Guercini et al. and Larson et al. also reported good results after treating 16 respectively 10 patients suffering from BPH with BTX–A [37, 38].

The doses used in intraprostatic injection of BTX–A are well below the presumed fatal dose and only minute quantities reach the systemic circulation. Dysuria and occasional minor hematuria were noted in three patients but the symptoms resolved by the next day [40]. Larson et al reported one case of acute epidydimitis [38]. The procedure is considered safe [39].

The longest follow–up data comes from Silva et al, who continuing the previous presented study [34] after 18 months had observed that mean prostate volume at baseline, 82± 16 ml progressively decreased from month one coming to 49± 9,5 ml ±p = 0,003) at month six, but from this moment on, prostate volume slowly recovered, becoming identical to baseline at 18 months ±73 ± 16 ml, p = 0.03).They did not observed the same trend in the maximal flow which remains relatively constant 11.3 ± 1.7 ml/sec [40]. 

The same favourable results are published also this year by Brisinda et al. [41] after injecting 200 UI BTX–A into prostate of 77 symptomatic patients. 41 patients had subjective symptomatic relief. Compared with baseline values, serum PSA was reduced from 6.2 ± 1.7 to 4.8 (1.0 ng/mL ±P = .03) and AUA score from 24.1 ± 4.6 to 12.6 ± 2.9 ±P = .00001). At the same time, prostatic volume and residual urine volume were reduced by 12.7% and 12.8%, respectively, and mean peak urinary flow rate increased ±P = .01). At 2 months' evaluation, 55 patients had subjective symptomatic relief. AUA score was reduced by 63.9% ±P=0.00001) compared with baseline values. In the same patients, serum PSA, prostatic volume, and residual urine volume were reduced by 51.6% ±P=0.00001), 42.8% ±P=0.00001), and 55.9% ±P=0.002), respectively, and mean peak urinary flow rate increased significantly [41] ±Table 1).

## Conclusion

Clinical series demonstrates efficacy of BTX–A in alleviating symptoms induced by BPH. Larger randomized clinical trials studies are necessary in order to identify the mechanisms by which BTX–A affects the prostate, the ideal dose and the duration of effect.
